# Macrophage exosomes modified by miR-365-2-5p promoted osteoblast osteogenic differentiation by targeting OLFML1

**DOI:** 10.1093/rb/rbae018

**Published:** 2024-02-24

**Authors:** Caiyao Hou, Yujue Zhang, Zhaoyong Lv, Yurun Luan, Jun Li, Chunxiu Meng, Kun Liu, Xin Luo, Liyu Chen, Fengzhen Liu

**Affiliations:** Department of Materials Science and Engineering, Liaocheng University, Liaocheng 252000, China; Liaocheng People’s Hospital, Liaocheng Hospital Affiliated Shandong First Medical University, Liaocheng 252000, China; Liaocheng People’s Hospital, Liaocheng Hospital Affiliated Shandong First Medical University, Liaocheng 252000, China; Liaocheng People’s Hospital, Liaocheng Hospital Affiliated Shandong First Medical University, Liaocheng 252000, China; Liaocheng People’s Hospital, Liaocheng Hospital Affiliated Shandong First Medical University, Liaocheng 252000, China; Liaocheng People’s Hospital, Liaocheng Hospital Affiliated Shandong First Medical University, Liaocheng 252000, China; Liaocheng People’s Hospital, Liaocheng Hospital Affiliated Shandong First Medical University, Liaocheng 252000, China; Liaocheng People’s Hospital, Liaocheng Hospital Affiliated Shandong First Medical University, Liaocheng 252000, China; The Second Hospital, Cheeloo College of Medicine, Shandong University, Jinan 250023, China; Department of Materials Science and Engineering, Liaocheng University, Liaocheng 252000, China; Liaocheng People’s Hospital, Liaocheng Hospital Affiliated Shandong First Medical University, Liaocheng 252000, China

**Keywords:** macrophage, exosomes, osteogenesis, osteoimmunology

## Abstract

In the bone immune microenvironment, immune cells can regulate osteoblasts through a complex communication network. Macrophages play a central role in mediating immune osteogenesis, exosomes derived from them have osteogenic regulation and can be used as carriers in bone tissue engineering. However, there are problems with exosomal therapy alone, such as poor targeting, and the content of loaded molecules cannot reach the therapeutic concentration. In this study, macrophage-derived exosomes modified with miR-365-2-5p were developed to accelerate bone healing. MC3T3-E1 cells were incubated with the culture supernatants of M0, M1 and M2 macrophages, and it was found that the culture medium of M2 macrophages had the most significant effects in contributing to osteogenesis. High-throughput sequencing identified that miR-365-2-5p was significantly expressed in exosomes derived from M2 macrophages. We incubated MC3T3-E1 with exosomes overexpressing or knocking down miR-365-2-5p to examine the biological function of exosome miR-365-2-5p on MC3T3-E1 differentiation. These findings suggested that miR-365-2-5p secreted by exosomes increased the osteogenesis of MC3T3-E1. Moreover, miR-365-2-5p had a direct influence over osteogenesis for MC3T3-E1. Sequencing analysis combined with dual luciferase detection indicated that miR-365-2-5p binded to the 3'-UTR of OLFML1. In summary, exosomes secreted by M2 macrophages targeted OLFML1 through miR-365-2-5p to facilitate osteogenesis.

## Graphical Abstract

**Figure:**
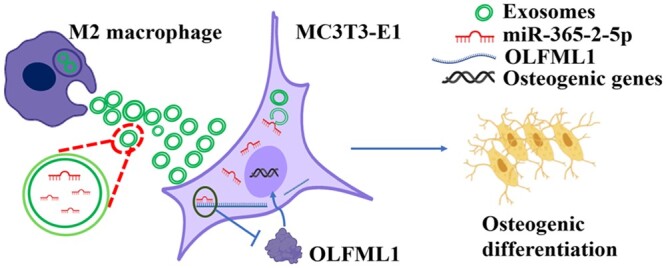

## Introduction

Various degrees of bone defects can result from bone tumors, trauma, osteomyelitis and osteonecrosis [1]. The inflammatory microenvironment mediated by immune cells is necessary for bone regeneration, while macrophages are major immunological effector cells with high malleability and are involved in various stages of bone restoration [2]. Macrophages are polarized into pro-inflammatory M1 and pro-tissue healing M2 phenotype, both of which are momentous for bone restoration [3]. M1 macrophages initiate the immune response involved in the acute inflammatory phase, while M2 macrophages perform the pivotal role in the later tissue healing phase. Prolongation and shortening of M1 and deficiency of M2 will exacerbate inflammatory damage [4]. In recent years, it has been increasingly demonstrated that macrophages regulate osteogenic activity by targeting mesenchymal stem cells via exosomes [5]. Exosomes are microvesicles (30–160 nm) secreted through the cell membrane and contain active molecules, such as microRNA (miRNA), lipid and protein of parental cell origin [6]. Thus, exosomes are nanomaterials of biological origin that serve as natural therapeutic carriers. They are mainly characterized by almost zero biotoxicity, high loading capacity and low immunogenicity [7]. In case of bone defects, exosomes secreted by macrophages act as nanocarriers to transmit signals within macrophages to osteoblasts, modulating the immune response while affecting the function of osteoblasts [8]. Therefore, exploring the specific mechanisms by which osteoblasts interact with M1 and M2 macrophages may be helpful in further guiding clinicians in the treatment of bone defect-related diseases.

MiRNAs are the main small non-coding RNAs in exosomes. The miRNAs are not only mediate the crosstalk between macrophages and osteoblasts in bone marrow microenvironment, but also in other tissue repairs [8–10]. The membrane of exosomes resists the unstable expression and degradation of miRNAs, so that exosome-encapsulated miRNAs are more biostable and can function precisely and efficiently [11, 12]. Exosomes transport miRNAs to osteoblasts and regulate skeletal function by pairing with the 3'-UTR of target genes causing degradation or translational repression of the genes [13]. As such, modification of miRNAs in macrophage exosomes could offer more efficacious treatment to bone defects. However, the most critical for bone regeneration is the sequential activation and timely conversion of the macrophage phenotype. Our previous findings suggested that exosomes derived from M1 and M2 macrophages contribute to BMSCs osteogenesis [14]. And further, miR-21a-5p derived from M1 macrophage exosomes can be transported to osteoblasts and target GATA2 to promote bone defect healing [15]. And yet, the mechanism of bone regeneration induced by M2 macrophage exosomes is still unclear.

The aim of this study was to investigate the effect of miRNA derived from M2 macrophage exosomes on osteogenic differentiation of mouse cranial osteoblasts (MC3T3-E1) and its potential therapeutic role in bone defect repair. With the insight of this process, we wish to prepare miRNA-modified M1 and M2 macrophage exosomes in combination with bone tissue engineering. To obtain biological substitutes that can remodel bone morphology and perform temporal immunomodulation to promote effective bone healing.

## Materials and methods

### Cell culture

MC3T3-E1 and murine-derived macrophage line RAW264.7 were purchased from American Tissue Culture Collection RAW264.7 cells and MC3T3-E1 cells were cultured in DMEM (10% fetal bovine serum (FBS) + 1% penicillin/streptomycin) and α-MEM (10% FBS + 1% penicillin/streptomycin), respectively.

### Effect of macrophages conditioned medium on osteogenic differentiation of MC3T3

#### Quantitative real-time polymerase chain reaction

RAW 264.7 cells were treated with 20 ng/ml interferon-γ (IFN-γ) and 100 ng/ml lipopolysaccharide (LPS) for 2 days to acquire M1 macrophages; 20 ng/ml interleukin-4 (IL-4) was used to treat RAW 264.7 to acquire M2 macrophages. Total RNA in cells was obtained with Trizol reagent, and reversed transcribed with a reverse transcription kit. The amplification pre-mixed solution and primers were added and amplified by PCR instrument. The primer sequences were listed in Supplementary Material S1. The relative expression of mRNA was calculated by 2^−△△Ct^ method.

#### Preparation of macrophages CM

M0, M1 and M2 macrophages were seeded in plates for 24 h. After that, the medium was removed and 3 ml DMEM was applied to wells. The culture supernatants of macrophages were collected after 24 h. The supernatants were mixed with α-MEM complete medium at a ratio of 1:1, and 50 μg/ml vitamin C, 10 mM β-glycerophosphate and 10 nM dexamethasone were added to prepare osteogenic induction solution [16]. They were named CM0, CM1 and M2 macrophage conditioned medium (CM2). The control group was α-MEM osteogenic induction medium.

### Influence of differently polarized macrophages CM on osteogenesis in MC3T3-E1

MC3T3-E1 osteoblasts were seeded in plates at 2 × 10^5^ cells/well, and 500 μl α-MEM was applied to wells. After 24 h, the α-MEM was replaced by the prepared osteogenic induction solution, and the solution was changed every 2 days. After 7 days, alkaline phosphatase (ALP) staining of MC3T3-E1 was performed. The expression of ALP, type I collagen (COL-1) and runt-related transcription factor 2 (Runx2) were examined by quantitative real-time polymerase chain reaction (qRT-PCR). Alizarin red S (ARS) staining of MC3T3-E1 was performed after 14 days.

#### ALP and ARS staining

The cells were washed twice with phosphate-buffered saline (PBS) and fixed with 4% paraformaldehyde at room temperature for 30 min, the residual liquid was removed and rinsed with PBS.

ALP staining was performed according to the instructions of BCIP/NBT Alkaline Phosphatase Color Development Kit (Beyotime, China) and incubate cells. ARS staining was performed by incubating cells with 0.02% ARS. All were carried out at room temperature. When the coloration reaches the desired depth, the reaction was terminated by removing the residual liquid and washing twice with PBS. Finally, the color depth was observed under the microscope and photographed. Quantitative analysis of ALP staining and ARS staining was performed using Image pro plus.

### Influence of M2 macrophage-derived exosomes on proliferation and osteogenic differentiation of MC3T3-E1

#### Isolation and identification of M2-exos

M2 macrophage-derived exosomes (M2-exos) were extracted by ultracentrifugation. The supernatant of M2 macrophage culture medium was centrifuged at 4°C (500 G for 10 min, 12 000 G for 20 min). After filtration through the 0.22 mm pore size filter and centrifugation at 100 000 G for 90 min, the upper layer was abandoned. The precipitate was suspended in PBS and continued to be centrifuged for 70 min. The precipitate was resuspended in PBS and stored at −80°C.

Identification of M2-exos by transmission electron microscope (TEM). Added 10 μl sample to the copper mesh to precipitate for 1 min, and absorbed the floating liquid. Uranium acetate (10 μl) was added to the copper mesh to precipitation for 1 min. The floating liquid was sucked off by filter paper, and dried at room temperature for several minutes. The morphology of exosomes was observed by imaging at 80–120 kV.

#### Endocytosis of the M2-exos by MC3T3-E1

Exosomes were stained and labeled according to the instructions of PKH67 (DLM, China). Exosomes were resuspended and diluted with α-MEM complete medium and added to the supernatant of MC3T3-E1 cell culture. After 24 h of culture, MC3T3-E1 was fixed with 4% paraformaldehyde for 40 min, and the membrane was broken by Triton X-100. The cells were covered with phalloidin solution at room temperature in the dark, and stained for 45 min. The nuclei were stained with DAPI.

#### The effect of M2-exos on proliferation of MC3T3-E1

MC3T3-E1 osteoblasts were seeded into plates. After cell adhesion, fresh medium was replaced, and exosomes with final concentrations of 0, 5, 15, 30 and 50 μg/ml were added. The group without exosomes was used as the control group. At 24 and 48 h, 100 μl of medium containing 10% Cell Counting Kit-8 (CCK-8) was placed into the wells. The absorbance at 450 nm was examined after 4 h.

#### Influence of M2-exos on osteogenesis in MC3T3-E1

MC3T3-E1 cells were seeded in 24-well plates at 2 × 10^5^ cells. After 24 h, the medium was changed to osteogenic induction medium containing 5 μg/ml of M2-exos. ALP staining and osteogenic gene expression were detected after 7 days of co-culture with MC3T3-E1. ARS staining was performed after 14 days.

### The effect of miR-365-2-5p derived from M2-exos on osteogenic differentiation of MC3T3-E1

#### MiRNA sequencing analysis

The miRNA sequencing analysis has been applied to identify miRNAs that were highly expressed in M2 exosomes. The specific methods were described as in previous studies [17]. Libraries were constructed with 500 ng of total RNA from M1 macrophage-derived exosomes (M1-exos) and M2-exos using Small RNA Sample Pre Kit. Illumina SE50 sequencing was performed after the test was qualified. The sequencing analysis was entrusted to Beijing Novogene Technology Co.

#### MiR-365-2-5p cell transfection

MiRNA-up lentivirus and miRNA-down lentivirus were purchased from Jikai biotechnology Co., Ltd. The RAW264.7 density reached 50%, miR-365-2-5p overexpression lentivirus (OE-miR-365-2-5p), overexpression negative control (OE-NC), miR-365-2-5p knockdown lentivirus and knockdown negative control were transfected into cells. After 48 h, green fluorescent protein (GFP) was detected. Subsequently, 2 mg/ml puromycin was added, and resistant cells were collected after 24–48 h incubation to obtain stable transfected cell lines. MC3T3-E1 cells were transfected with miR-365-2-5p overexpressing lentivirus and negative control (NC) in the same way.

### The effect of miR-365-2-5p derived from M2 macrophage exosomes on osteogenesis in MC3T3-E1

The RAW264.7 transfected with lentivirus was induced into M2 type, and exosomes were extracted. MC3T3-E1 cells were seeded in 24-well plates at a density of 5 × 10^3^ cells/well. After 24 h, exosomes were added to the α-MEM osteogenic induction medium. ALP staining and osteogenic gene expression were detected after 7 days of co-culture. ARS staining was detected 14 days later.

#### Influence of miR-365-2-5p on proliferation and osteogenesis in MC3T3-E1

MC3T3-E1 osteoblasts were transfected with miR-365-2-5p overexpression and negative expression lentivirus. Afterwards, CCK-8 was used to measure cell proliferation. Two groups of MC3T3-E1 osteoblasts were seeded in 24-well plates. After 24 h, it was replaced by α-MEM osteogenic induction solution. After 7 days, ALP staining was examined. Furthermore, mRNA and protein expression of osteogenesis-related molecules were detected. ARS staining was performed 14 days later.

#### Western blot

After 7 days of cell culture in each group, the cells were lysed by Radio Immunoprecipitation Assay to extract total proteins, and the protein concentration was estimated by Enhanced BCA Protein Assay Kit (Beyotime, China). The same amount of sample protein was added to a 10% SDS-PAGE well and transferred to a hydrophobic Polyvinylidene Fluoride (PVDF) membrane. After blocking the membrane antibody with 8% skim milk for 60 min, it was immersed in the primary antibody solution of Runx2, BMP2 and OPN (Beyotime, China) at a ratio of 1:1000 and overnight at 4°C. The membrane was washed three times with quickblock™ blocking buffer (Beyotime, China), and then incubated with secondary antibody solution of HPR-labeled goat anti-rabbit IgG (Beyotime, China) at a ratio of 1:1000. The PVDF membrane was washed with quickblock™ blocking buffer and then reacted with ECL developing reagent. The bands were exposed by chemiluminescence gel imaging system (Bio-Rad, USA).

### Mechanism of miR-365-2-5p secreted by M2-exos to promote MC3T3-E1 osteogenic differentiation

#### High-throughput sequencing and bioinformatics analysis

Total RNA was extracted after MC3T3-E1 transfection of overexpressing miR-365-2-5p lentivirus and NC lentivirus. Total amounts and integrity of RNA were assessed using the RNA Nano 6000 Assay Kit of the Bioanalyzer 2100 system (Agilent Technologies, CA, USA). Subsequently, the library was prepared. Finally, the library was sequenced by Illumina NovaSeq 6000. Differential gene (DE) analysis by DESeq2 R package (1.20.0).

#### Dual luciferase reporter gene assay

Two hundred and ninety-three T cells were transfected with miR-365-2-5p overexpression lentivirus (OE-miR-365-2-5p) and negative expression lentivirus (OE-NC). The normal 3'-UTR sequence (WT) and point mutation 3'-UTR sequence (MT) of OLFML1 were inserted into the pmir GLO vector to construct a dual luciferase reporter vector. WT-OLFML1 and MT-OLFML were transfected into 293 T-OE-miR-365-2-5p cells and 293 T-OE-NC cells by X-tremeGENE 9 Reagent (Roche, USA), respectively. The intracellular renilla and firefly fluorescence activity were determined by a dual luciferase reporter gene detection system after 48 h. The fluorescence value of the reporter gene = firefly fluorescence value/renilla fluorescence value.

### Statistical analysis

All data were treated with GraphPad Prism 8 statistical software. Two-tailed Student’s *t*-test were applied for comparison among the two groups, and one-way ANOVA was performed for comparisons among several groups. *P* < 0.05 was considered statistically significant.

## Results

### M2 macrophage conditioned medium promotes osteogenesis in MC3T3-E1

**Figure 1.**
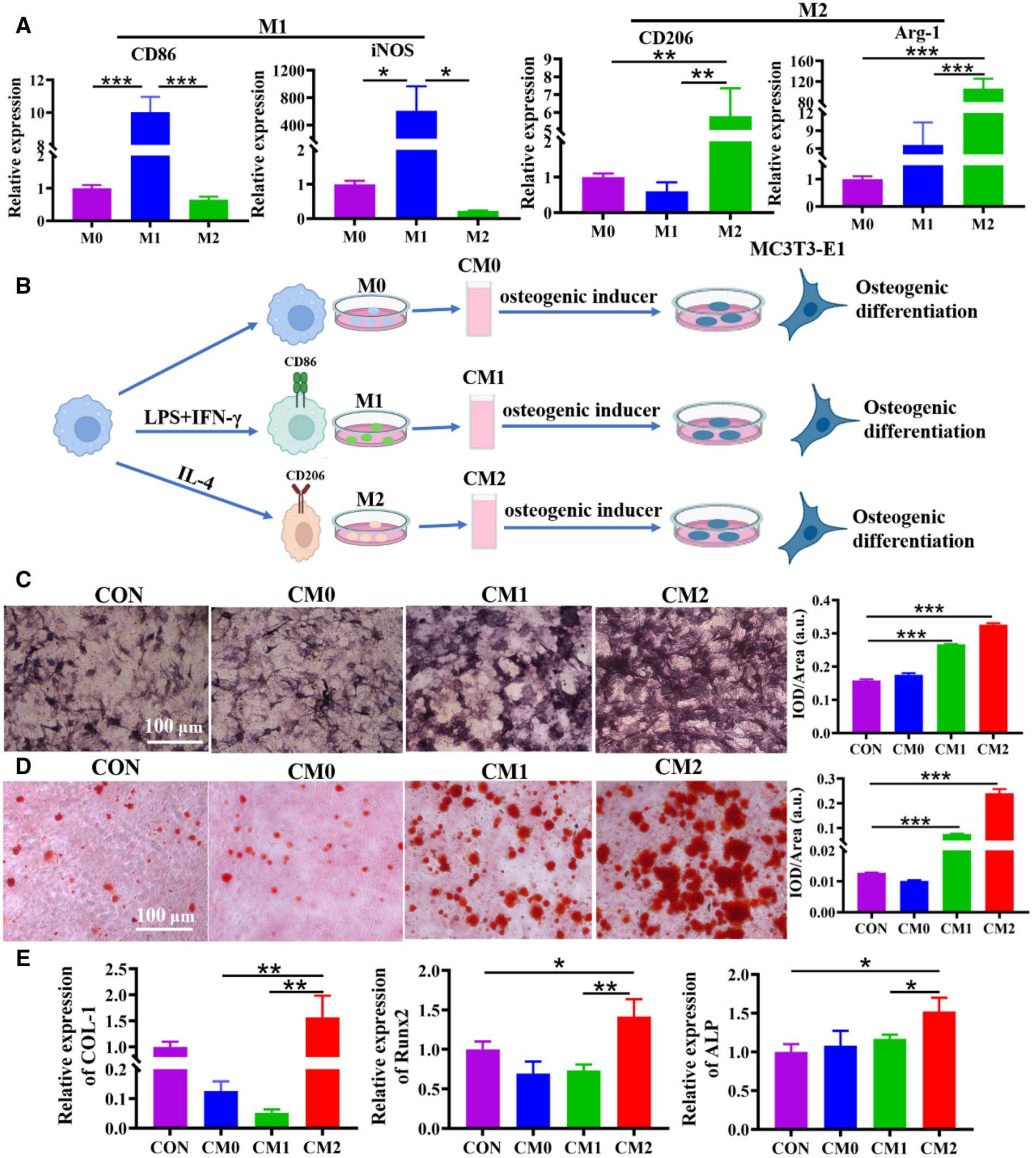
Effects of conditioned medium on MC3T3-E1 osteogenesis. (**A**) The expression of CD86 (M1 marker) and CD206 (M2 marker) in macrophages was measured by qRT-PCR. (**B**) The schematic diagram of co-culture for macrophages and MC3T3-E1. (**C**) The osteogenesis of MC3T3-E1 was analyzed by ALP staining after 7 days in the co-culture system. (**D**) The ability of MC3T3-E1 to form mineralized nodules was analyzed by ARS staining after 14 days in the co-culture system. (**E**) The mRNA expression of COL-1, Runx2 and ALP in MC3T3-E1 was analyzed by qRT-PCR after 7 days in the co-culture system. ^*^P<0.05, ^**^P<0.01, ^***^P<0.01.

Macrophages have plasticity, and different microenvironment signal stimulation can significantly change their biological functions. RAW264.7 macrophages (M0) polarized to M1 and M2 types following induction by suitable cytokines. The mRNA expression of macrophage subtype markers was examined by qRT-PCR. The findings indicated that M1 marker CD86, iNOS was positively expressed in macrophages induced by LPS and IFN-γ, and M2 marker CD206, Arg-1 was positively expressed in macrophages induced by IL-4 (Figure 1A). In order to identify the influence of macrophages polarization on osteogenesis of MC3T3-E1, a co-culture system of macrophages and MC3T3-E1 was constructed (Figure 1B). Compared to controls, CM2 significantly increased ALP staining, mineralized nodule formation and osteogenesis-related expression (ALP, COL-1 and Runx2) in MC3T3-E1 (Figure 1C–E).

### M2-exos promote the proliferation of MC3T3-E1 cells

**Figure 2.**
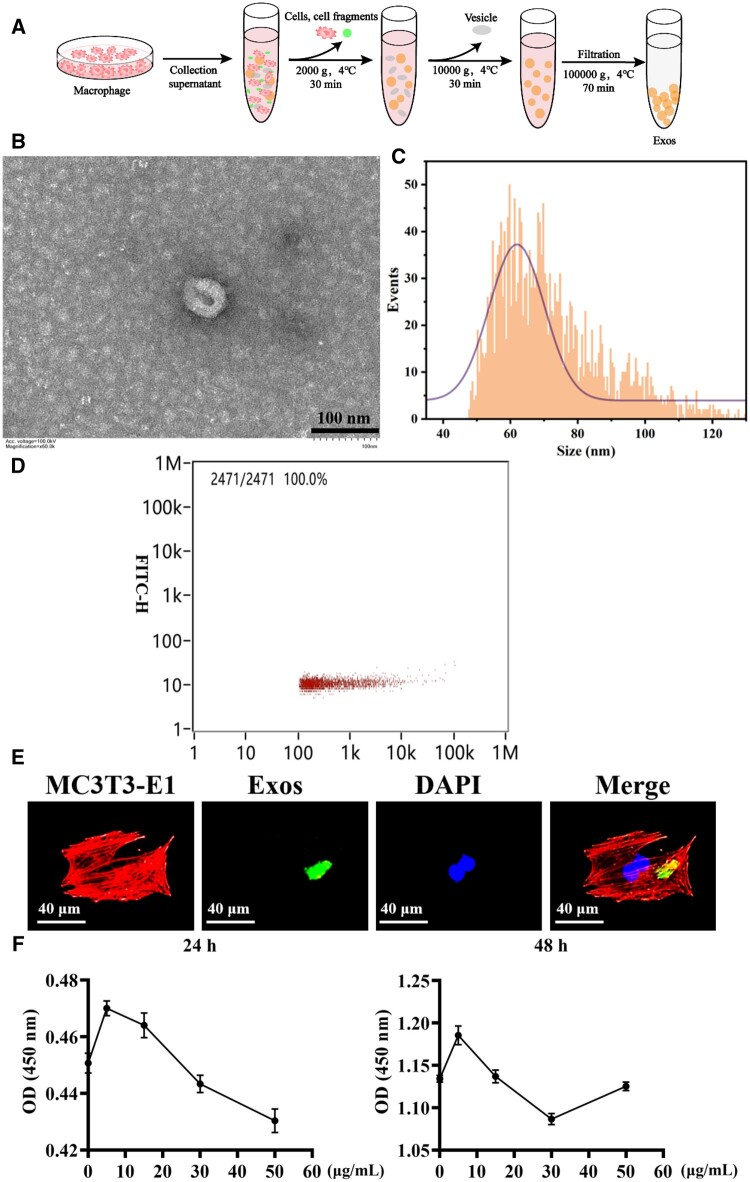
Extraction and characterization of exosomes secreted by M2 macrophage. (**A**) Exosomes preparation process. (**B**) TEM diagram of exosomes (scale bar: 100 nm). (**C**) Particle size analysis of exosomes. (**D**) Concentration information of exosomes. (**E**) Analysis of exosome uptake by MC3T3-E1. (**F**) Effects of different concentrations of M2-exos on MC3T3-E1 proliferation.

Exosomes are extracellular microvesicles secreted by cells and are an important way of intercellular communication. Next, exosomes in the supernatant of M2 medium were extracted by differential centrifugation (Figure 2A). Transmission electron microscopy showed that the exosome was oval vesicles with a diameter of about 150 nm (Figure 2B). Particle size and concentration analyses using a nanoparticle size analyzer. The results showed that the average particle size was 71.75 nm and the concentration was 2.08E + 10 particles/ml (Figure 2C and D). To explore the cellular uptake of exosomes, MC3T3-E1 with PKH 67 labeled exosomes were co-cultured. As shown in Figure 2E, the blue and red fluorescence were the nucleus and cytoskeleton of MC3T3-E1, respectively, while the green fluorescence was exosomes. The figure showed the presence of green fluorescence in the recipient cells, suggesting that exosomes were internalized into the cytoplasm and further utilized by the cell. It had been found that drugs promoted the proliferation of osteoblasts to promote bone formation [18]. Then, the impact of different concentrations of M2 macrophage exosomes on cell proliferation was examined. After co-culture of exosomes and cells for 24 and 48 h, 5 μg/ml exosomes enhanced the proliferation of MC3T3-E1 most effectively (Figure 2F).

### Differential miRNAs expression in M2-exos

**Figure 3.**
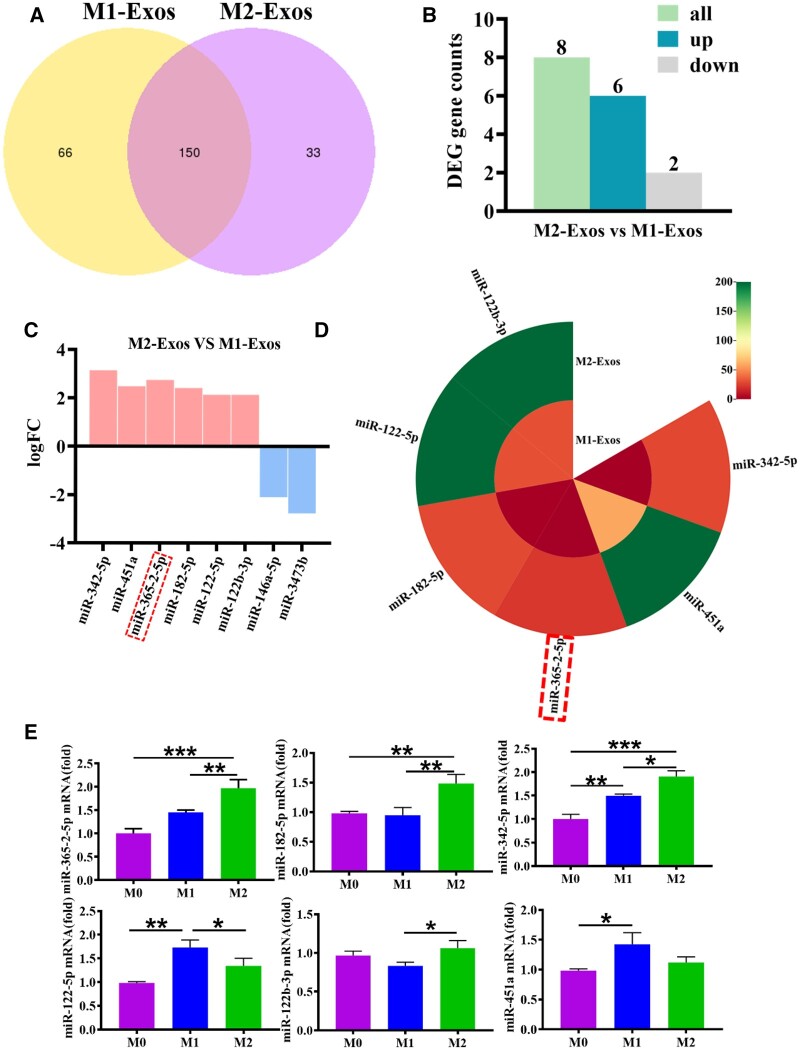
Identification of differentially expressed miRNAs of M2-exos. (**A**) Expression of miRNAs in M1-exos and M2-exos. (**B**) The number of differentially expressed miRNAs in M2-exos. (**C**) Differentially expressed miRNAs in M2-exos. (**D**) Expression profiles of differential miRNAs highly expressed in M2-exos. (**E**) Differential miRNAs highly expressed in M2-exos were confirmed by qRT-PCR.^*^P<0.05, ^**^P<0.01, ^***^P<0.001.

Previously, M0, M1 and M2 exosomes were extracted and co-cultured with osteoblasts, respectively, and it was found that M2 exosomes promoted osteogenic differentiation [8]. Therefore, the mechanism of M2 exosomes promoting osteogenesis was explored. Total RNA from M1-exos and M2-exos were obtained for high-throughput sequencing. The Venn diagram showed that 216 miRNAs were expressed in M1-exos and 183 miRNAs were expressed in M2-exos, with a total of 150 miRNAs (Figure 3A). A total of eight miRNAs were screened in M2-exos compared to M1-exos, of which six miRNAs were up-regulated and two miRNAs were down-regulated (Figure 3B). The differential miRNA screening condition was padj < 0.05 and |log2(foldchange)| (|log2FC|) > 1. As shown in Figure 3C, miR-342-5p, miR-451a, miR-365-2-5p, miR-182-5p, miR-122-5p and miR-122b-3p were overexpressed in M2-exos. In addition, miR-365-2-5p was not expressed in M1-exos (Figure 3D). The overexpressed miRNAs in M2-exos were confirmed by qRT-PCR. The findings indicated that miR-365-2-5p, miR-182-5p and miR-342-5p were consistent with the sequencing results (Figure 3E). In particular, miR-182-5p and miR-342-5p negatively regulated osteogenic differentiation while miR-365 promoted bone formation [19–21]. The results of qRT-PCR showed that although miR-365-2-5p was expressed in M0 and M1 exosomes, the expression level was low and not significant. Combined with sequencing results, it was found that miR-365-2-5p could not be stably expressed in M1 exosomes. Therefore, we investigated the effect of miR-365-2-5p in M2-exos on MC3T3-E1 osteogenesis.

### MiR-365-2-5p secreted by M2-exos promoted osteogenesis of MC3T3-E1

Next, the effect of miR-365-2-5p secreted by M2-exos on osteogenic differentiation of MC3T3-E1 was investigated. Firstly, M2-exos that overexpressed and knocked down miR-365-2-5p were prepared. As shown in Figure 4A, lentivirus was successfully transfected into macrophages. Macrophages were induced to M2 type, and the expression of miR-365-2-5p was detected by qRT-PCR. The findings indicated that miR-365-2-5p was significantly overexpressed or knocked down in M2-exos (Figure 4B). Subsequently, M2 macrophage exosomes (overexpressing or knocking down miR-365-2-5p) were co-cultured with MC3T3-E1 to detect the effect of miR-365-2-5p in MC3T3-E1. As shown in Figure 4C, the trend of miR-365-2-5p in MC3T3-E1 was consistent with that in M2 macrophage exosomes. Finally, the influence of exosomal miR-365-2-5p on MC3T3-E1 osteogenesis was explored by adding M2-exos that overexpressed and knocked down miR-365-2-5p during MC3T3-E1 osteogenic differentiation. Compared with the NC group, ALP staining was remarkably enhanced, the number of calcified nodule formation was apparently increased, and the expression of osteogenesis-related genes was enhanced in the M2-OE-miR-365-2-5p group. In the M2-KD-Exo-miR-365-2-5p group, ALP staining was attenuated, the number of calcified nodule formation was clearly reduced, and miRNA expression of ALP, Runx2 and BMP2 was diminished (Figure 4D–F).

**Figure 4.**
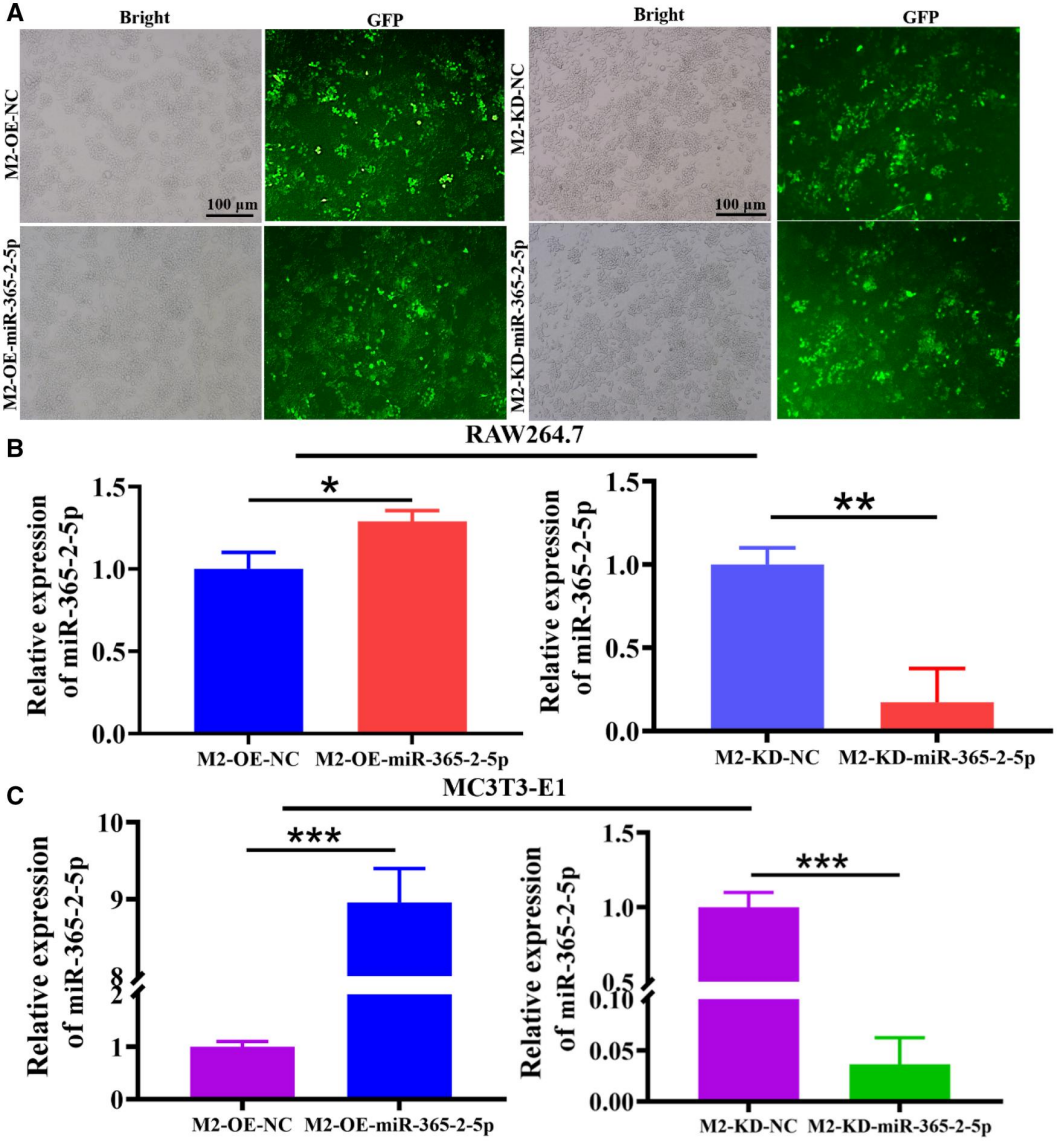
Influence of miR-365-2-5p secreted by M2-exos on MC3T3-E1 osteogenesis. (**A**) Fluorescence microscopy for GFP expression. (**B**) MiR-365-2-5p expression in M2 macrophage was confirmed by qRT-PCR. (**C**) Expression of miR-365-2-5p in cells following co-culture of MC3T3-E1 with M2-exos overexpressing and knocking down miR-365-2-5p by qRT-PCR. (**D**) ALP staining to detect the impact of M2-exos secreted miR-365-2-5p on MC3T3-E1 osteogenic differentiation (scale bar: 100 µm). (**E**) ARS staining was employed to observe the influence of miR-365-2-5p secreted by M2-exos on MC3T3-E1 mineralization (scale bar: 100 µm). (**F**) Influence of miR-365-2-5p secreted by M2-exos on the expression of osteogenesis-related genes (ALP, Runx2 and BMP2) in MC3T3-E1 cells. ^*^P<0.05, ^**^P<0.01, ^***^P<0.001.

**Figure 4.**
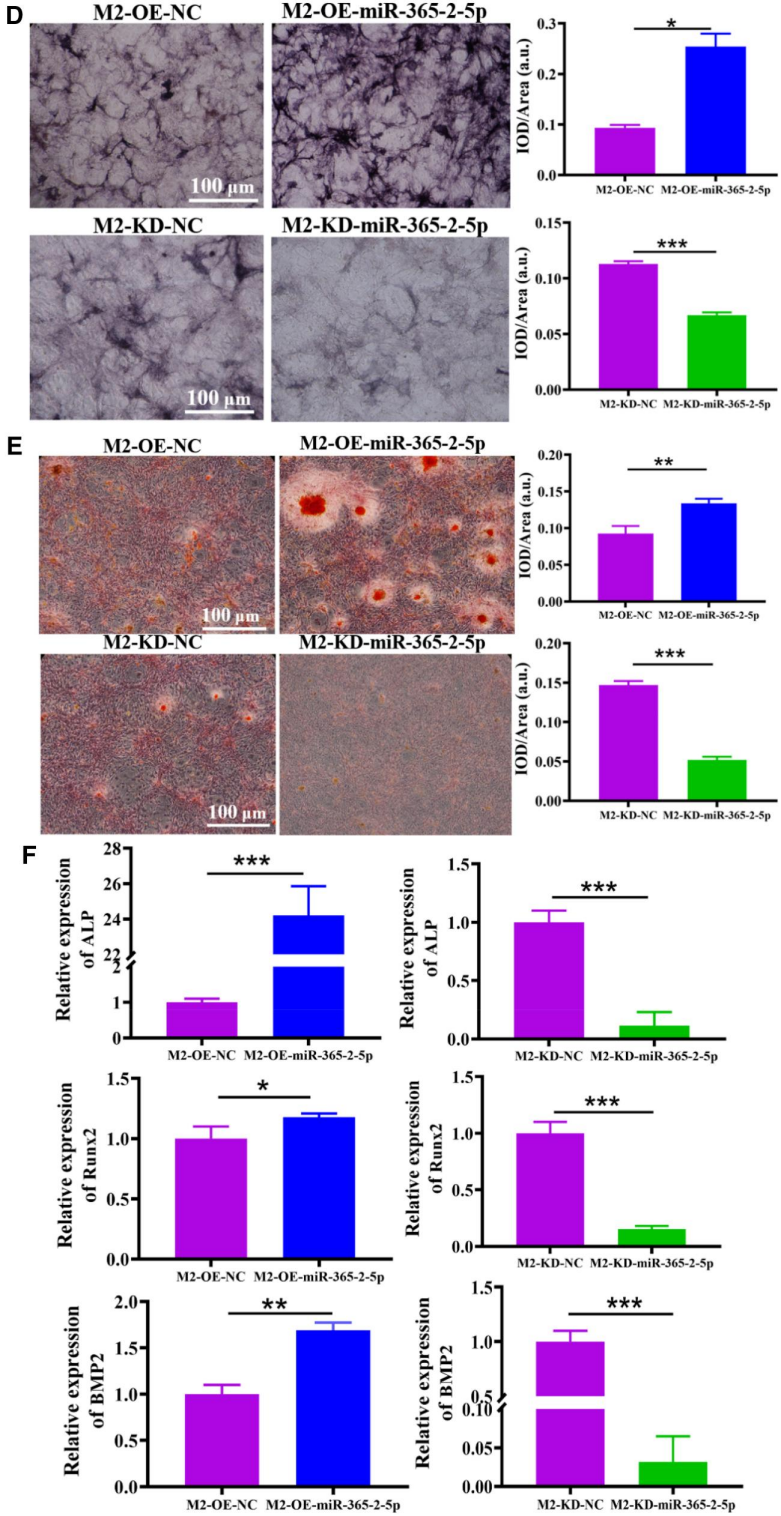
(Continued).

### MiR-365-2-5p promoted osteogenic differentiation of MC3T3-E1

**Figure 5.**
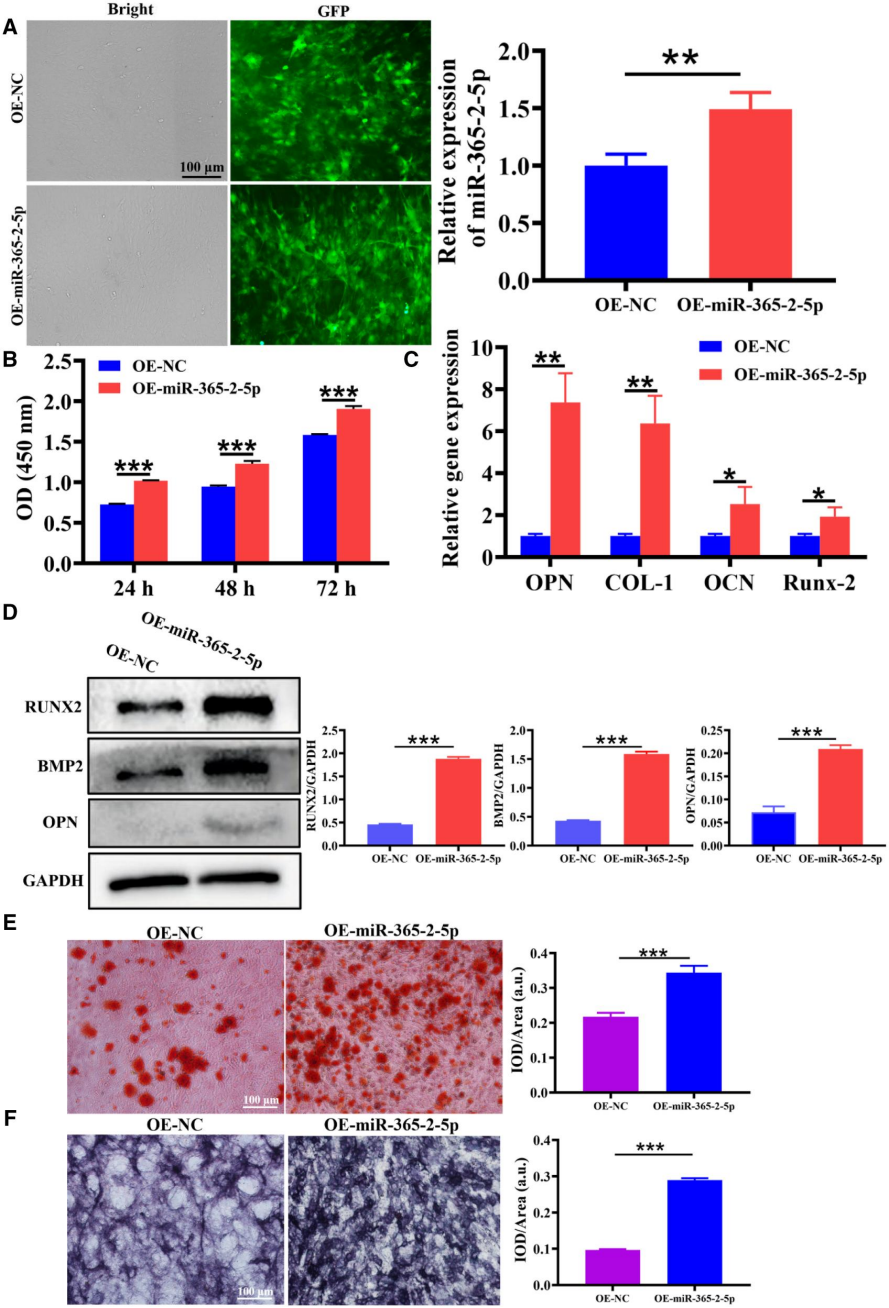
The effect of miR-365-2-5p on osteogenic differentiation of MC3T3-E1. (**A**) A cell model of MC3T3-E1 overexpressing miR-365-2-5p was constructed. (**B**) CCK-8 was used to examine the efficacy of miR-365-2-5p on the proliferation of MC3T3-E1. (**C**) The effects of miR-365-2-5p on mRNA expression of OPN, COL-1, OCN and Runx2 were measured by qRT-PCR. (**D**) The influence of miR-365-2-5p on Runx2, BMP2 and OPN protein expression was measured by WB. (**E**) The influence of miR-365-2-5p on extracellular matrix mineralization was observed by ARS staining (scale bar: 100 µm). (**F**) The influence of miR-365-2-5p on osteogenesis of MC3T3-E1 was observed by ALP staining (scale bar: 100 µm). ^*^P<0.05, ^**^P<0.01, ^***^P<0.001.

Subsequently, we investigated the capability of miR-365-2-5p to directly influence MC3T3-E1 osteogenesis. As displayed in Figure 5A, the expression of miR-365-2-5p in MC3T3-E1 was greatly increased. The CCK-8 kit was intended for the examination of cell proliferation. The results indicated that overexpression of miR-365-2-5p (OE-miR-365-2-5p) promoted MC3T3-E1 proliferation (Figure 5B). In addition, miRNA expression of OPN, COL-1, OCN and Runx2 was examined by qRT-PCR, which indicated a clear increase in the OE-miR-365-2-5p group (Figure 5C). The protein expression of Runx2, BMP2 and OPN was detected by Western blot (WB) and found to be notably increased in the OE-miR-365-2-5p group (Figure 5D). To research the effect of miR-365-2-5p for extracellular matrix mineralization, ARS staining was performed following 14 days of MC3T3-E1 osteogenic induction revealing increased mineral deposition in the OE-miR-365-2-5p group (Figure 5E). As well, miR-365-2-5p evoked enhanced ALP staining (Figure 5F).

### The mechanism of miR-365-2-5p-mediating MC3T3-E1 osteogenic differentiation was analyzed by bioinformatics

**Figure 6.**
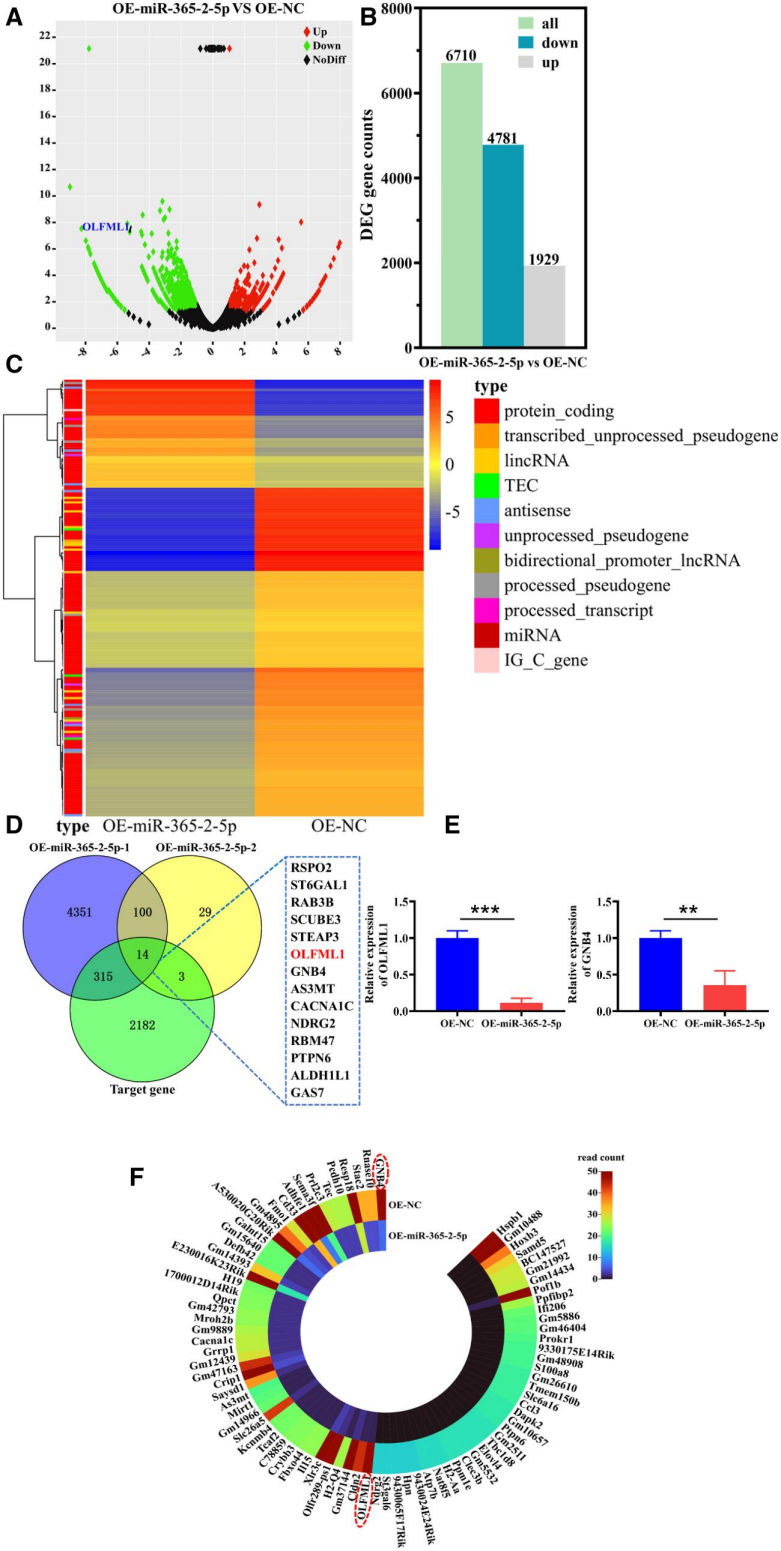
The mechanism of miR-365-2-5p mediating MC3T3-E1 osteogenic differentiation was analyzed by bioinformatics. (**A**) Volcano map of DEs in OE-NC group and OE-miR-365-2-5p group. (**B**) Histogram of DEs in OE-NC and OE-miR-365-2-5p groups. (**C**) Heatmap of DEs in OE-NC and OE-miR-365-2-5p groups. (**D**) The target genes of miR-365-2-5 p were analyzed by Venn diagram. (**E**) The expression of OLFML1 and GNB4 in MC3T3-E1 cells was determined by qRT-PCR. (**F**) Heatmap of down-regulated gene expression profiles in the OE-miR-365-2-5p group. ^**^P<0.01, ^***^P<0.001.

To explore the mechanism by which miR-365-2-5p promoted MC3T3-E1 osteogenesis, we extracted the miRNA of MC3T3-E1 in OE-NC group and OE-miR-365-2-5p group for high-throughput sequencing analysis. Compared to the OE-NC group, the OE-miR-365-2-5p group had 6710 DEs, of which 4781 genes were down-regulated and 1929 genes were up-regulated (Figure 6A and B). Padj ≤ 0.05 and |log2FC| ≥ 1 were set as the threshold for significantly differential expression. The heat map of cluster analysis showed that the two groups were mainly different in protein coding (Figure 6C). The down-regulated genes and the miR-365-2-5p target genes predicted by the TargetScan database were intersected, ultimately 14 candidate genes were identified (Figure 6D). Literature research found that only OLFML1 and GNB4 negatively regulated osteogenic differentiation [22, 23]. Therefore, the mRNA expression of OLFML1 and GNB4 was verified by qRT-PCR. The decrease of OLFML1 in the OE-miR-365-2-5p group was more significant compared to the GNB4 (Figure 6E). This was consistent with the results of gene expression profiles (Figure 6F).

### MiR-365-2-5p promoted MC3T3-E1 bone formation by targeting OLFML1

**Figure 7.**
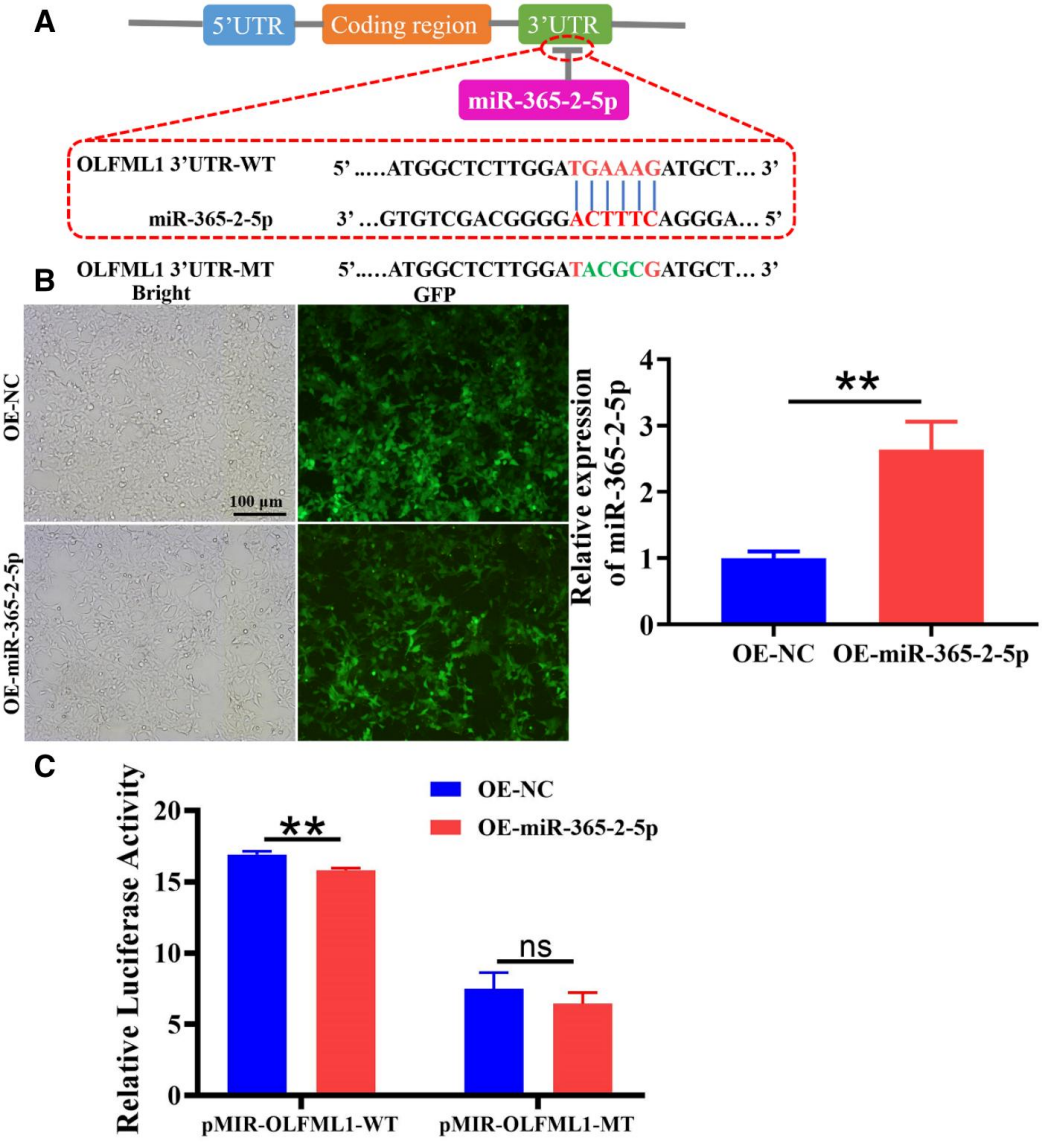
MiR-365-2-5p Promoted MC3T3-E1 bone formation by targeting OLFML1. (**A**) Diagram of miR-365-2-5p and OLFML1 binding sequence. (**B**) The construction of 293 T cell model overexpressing miR-365-2-5p. (**C**) The targeting relationship between miR-365-2-5p and OLFML1 was detected by dual luciferase assay.^**^P<0.01.

Ultimately, the regulatory mechanism of miR365-2-5p and OLFML1 was evaluated. The targetscan database predicted a binding site between miR365-2-5p and OLFML1 (Figure 7A). After transfection of 293 T cells with miR-365-2-5p overexpression lentivirus and empty lentivirus, OLFML1-3'-UTR-WT plasmid and OLFML1-3'-UTR-MT plasmid were transfected, respectively, for luciferase reporter gene activity detection (Figure 7B). The outcomes indicated that overexpression of miR-365-2-5p reduced the fluorescence expression of OLFML1-3'-UTR-WT, and had no remarkable impact of OLFML1-3'-UTR-MT (Figure 7C).

## Discussion

The essence of bone regeneration is a specific type of foreign body reaction that involves a series of resident cell/factor [24]. It has been shown that the immune system influences the course and outcome of bone healing by regulating the switch from an inflammatory to an anti-inflammatory phenotype of cells [25]. Therefore, we consider whether it is possible to promote bone healing by modulating immunity. Among congenital immune cells, the macrophage is considered to be a key factor in coordinating the reconstruction of tissue function after injury [26].

Depending on the local bone microenvironment, macrophages can be polarized into either classically activated (M1) and alternatively activated (M2) phenotypes [27]. M1 macrophages can be activated by LPS or pro-inflammatory cytokines, participate in host defense processes, and highly express iNOS and CD86 [28]. M2 macrophages can be activated by IL-4, play a role in promoting tissue repair and highly express receptor molecules Arg-1 and CD206 [29]. In this research, we successfully induced macrophages polarization into M1 phenotype and M2 phenotype. And macrophages in different phenotypes were co-cultured with MC3T3-E1 to detect their effects on MC3T3-E2 osteogenic differentiation. ALP was an important phosphatase that promoted bone formation. The formation of calcified nodules was a unique hallmark of osteoblasts. Runx2 and COL-I were considered important biomarkers in bone formation [30, 31]. ALP staining, ARS staining and gene expression of ALP, Runx2 and COL-I suggested that M2 macrophages promoted osteogenic differentiation of MC3T3-E1 (Figure 1).

Exosomes were vesicles with a diameter of 30–200 nm released by cells into the extracellular space and an important application in tissue engineering was as a nanocarrier for loading proteins, nucleic acids and drugs. Exosomes entered target cells mainly through membrane fusion, and the signaling molecules they carried acted on a variety of signaling pathways in target cells to participate in intercellular information transfer [32, 33]. Therefore, the internalization of exosomes by target cells was the basis and premised for their biological function regulation of target cells. This study found that M2-exos was internalized by MC3T3-E1 and 5 μg/ml M2-exos dramatically promoted MC3T3-E1 proliferation (Figure 2).

It had been reported that miRNAs were involved in osteogenic differentiation of various cells [34]. Hu *et al.* [35] found that miR-1224-5p promoted osteoblast differentiation by targeting ADCY2 through the rap1 signaling pathway. Huang *et al.* [36] demonstrated that the exosomal miR-19b sourced from mesenchymal stem cells augments osteogenesis of BMSCs *via* the WWP1/Smurf2-mediated KLF5/β-catenin signaling pathway. In this study, miR-365-2-5p was notably overexpressed in M2-exos and promoted osteogenic differentiation (Figure 3). Exosomal molecules with a specific function were unstable in content and low in homogeneity. Therefore, it was of great clinical importance to modify exosomes. In this study, miR-365-2-5p-modified M2-exos were co-cultured with MC3T3-E1 to investigate the effect of miR-365-2-5p in exosomes. The findings indicated that miR-365-2-5p was translocated to MC3T3-E1 *via* M2-exos and promoted osteogenic differentiation of MC3T3-E1 (Figure 4). Moreover, miR-365-2-5p can directly enhance the osteogenic ability of MC3T3-E1 (Figure 5).

The role of miR-365-2-5p derived from M2-exos in mediating osteogenesis of MC3T3-E1 prompted us to discover the mechanisms related to the event. It was found that miR-365-2-5p inhibited the expression of OLFML1 (Figure 6). A study had shown that mutation of OLFML1 lead to impaired osteoblast differentiation and abnormal development of bone tissue [22]. The dual luciferase gene reporting technology verified the targeting relationship between miRNA and OLFML1. It was suggested that miR-365-2-5p can specifically bind to OLFML (Figure 7).

**Figure 8.**
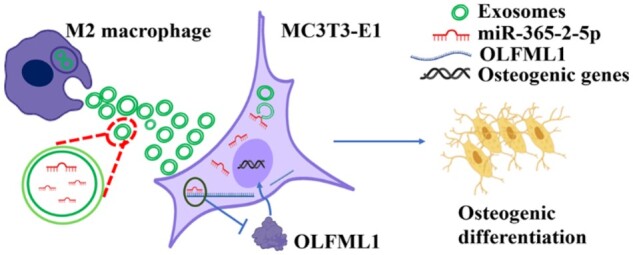
MiR-365-2-5p derived from M2 macrophage exosomes promotes osteogenesis of MC3T3-E1 by targeting OLFML1.

In summary, this study provided evidence that M2-exos can promote osteogenesis of MC3T3-E1 through miR-365-2-5p. At the same time, the event involved the downregulation of OLFML1 (Figure 8). However, this study did not involve evidence related to the effect of miR-365-2-5p derived from M2-exos on bone defect repair *in vivo*. Moreover, bone metabolism is a continuous and dynamic remodeling process whose homeostatic depends on the dynamic balance between osteoclast and osteoblast composition. It remains questionable whether M2-exos target osteoclasts and inhibit osteoclast overproduction. In future studies, we will observe the effects of miR-365-2-5p derived from M2-exos on new bone production in rats and focus on molecular mechanisms related to M2-exos-mediated osteoclasts. In addition, exosomes have a short half-life *in vivo* and are easily cleared by the immune system [37]. Therefore, the development of tissue-engineered scaffolds for the delivery of exosomes is an interesting research direction.

## Conclusion

In conclusion, miR-365-2-5p derived from M2 macrophage exosomes promotes osteogenesis of MC3T3-E1 by targeting OLFML1. The current findings provide new insights in clinical practice for treating bone defects with exosomes. The sequential release of miRNA-modified M1 and M2 macrophage exosomes from bone tissue-engineered scaffolds will be a possible way to treat diseases, such as bone defects in the future.

## Supplementary Material

rbae018_Supplementary_Data

## Data Availability

All datasets are obtainable from the corresponding author at fair demand.
