# Supplementary material for: Macrophage exosomes modified by miR-365-2-5p promoted osteoblast osteogenic differentiation by targeting OLFML1
Source: Regen Biomater. 2024 Feb 24;11:rbae018. doi: 10.1093/rb/rbae018 (PMC10939467; doi:10.1093/rb/rbae018)
Supplement: rbae018_Supplementary_Data [file rbae018_supplementary_data.docx]

Supplementary 1 Primer sequences

F: Forward, R: Reverse.
